# High PHD Finger Protein 19 (PHF19) expression predicts poor prognosis in colorectal cancer: a retrospective study

**DOI:** 10.7717/peerj.11551

**Published:** 2021-06-01

**Authors:** Pengfei Li, Jie Sun, Yuanyuan Ruan, Lujun Song

**Affiliations:** 1 Department of General Surgery, Zhongshan Hospital, Fudan University, Shanghai, China; 2 Department of Biochemistry and Molecular Biology, School of Basic Medical Sciences, Fudan University, Shanghai, China; 3 Cancer Center, Zhongshan Hospital, Fudan University, Shanghai, China

**Keywords:** Colorectal cancer (CRC), PHF19, Prognosis

## Abstract

**Background:**

Colorectal cancer (CRC) is the third most common cancer all around the world, and it seriously threats human health. PHF19 has been proved to be closely related to the prognosis of patients in a variety of malignant tumors, but the effect of PHF19 on the prognosis evaluation of CRC patients has not been confirmed.

**Methods:**

In our study, we used GEO, TCGA database and IHC to verify the PHF19 expression in CRC samples. Survival analysis of PHF19 based on TCGA, GEO series, and our own CRC sample were performed. Cox regression was performed to reveal the relationship between PHF19 and prognosis. Co-expression was performed to find genes related to PHF19 expression. GO/KEGG enrichment analysis and GSEA analysis were used to confirm the most relevant signal pathway to PHF19. Next, cell experiments were performed to verify the effect of PHF19 on the proliferation, invasion and metastasis of CRC. Then, Western blot was used to verify the protein expression of the above two phenotypes. Finally, tumor formation experiments in nude mice were used to verify the role of PHF19 of tumor proliferation in vivo.

**Results:**

We found that PHF19 was significantly over-expressed in tumors compared with normal tissues. Kaplan–Meier (K–M) analysis indicated that high PHF19 in CRC associated with poor overall survival (OS) in CRC patients. Clinical correlation analysis showed that high expression of PHF19 was closely related to t umor progression in CRC patients, especially infiltration and metastasis. Bioinformatics revealed that PHF19 might affect tumor malignant phenotype by regulating the cell cycle in CRC. CCK-8 and clonal formation experiment showed that the proliferative ability of tumor cells was promoted. Flow cytometry showed that the cell cycle accelerated the transition from G1 to S phase. Western blot found that Cyclin D1, CDK4, and CDK6 expression were up-regulated. Transwell and wound-healing experiment found that invasive and migratory abilities was promoted after the over-expression of PHF19. Western blot showed that the expression of key proteins of Epithelial-Mesenchymal Transition (EMT) changed. Tumor formation experiments in nude mice showed that overexpression of PHF19 could promote tumor proliferation in vivo.

**Conclusion:**

Our research proved that PHF19 could be an independent prognostic factor for CRC, PHF19 promoted the proliferative ability and the invasion and metastasis of CRC by up-regulating the expression of key molecules related to cell cycle and EMT pathway in vitro, promoting tumor proliferation in vivo.

## Introduction

Colorectal cancer (CRC) is the third most common cancer all around the world, and it seriously threatens human health (Bray et al., 2018; Weitz et al., 2005). According to official statistics, there were nearly 1.8 million newly diagnosed patients with CRC and more than 800,000 deaths, which accounted for 10% of the number of newly diagnosed cancers worldwide in 2018 (Bray et al., 2018). In recent years, diagnosis and treatment methods have made great progress, and the rise of targeted therapy based on molecular biology research has brought new hope to CRC patients (Molgora et al., 2020; Roncucci & Mariani, 2015). Although there are many technologies such as surgery, radiotherapy, chemotherapy, and molecular targeted therapy, some patients still suffer from the pain of cancer recurrence and further progress after surgery (Chen et al., 2020; Zhang et al., 2020). Current research has confirmed that many genes are closely related to the occurrence and development of CRC, but the genetic research related to the occurrence and development of CRC is far from enough.

Polycomb family (PcG) is a type of transcription factor that regulates target genes through epigenetic modification at the chromatin level, which usually exists in the form of a Polycomb protein complex (PRC) (Deng et al., 2018; Sauvageau & Sauvageau, 2010). PRC is usually divided into two types, PRC1 and PRC2, which play important functions in cell proliferation and differentiation, embryonic development, and tumorigenesis (Brien et al., 2012; Cai et al., 2018; Ren et al., 2019; Sauvageau & Sauvageau, 2010). PHD Finger Protein 19 (PHF19), also known as PCL3, is a member of the PRC2 complex (Qin et al., 2013). PHF19 is usually involved in the activation of chromosomes to perform biological functions and it has recently been confirmed to be closely related to the occurrence and development of tumors (Ballare et al., 2012; Brien et al., 2012; Cai et al., 2018; Deng et al., 2018; Qin et al., 2013; Wang, Robertson & Zhu, 2004). In liver cancer, glioblastoma, multiple myeloma, gastric cancer, and melanoma, PHF19 has been confirmed to be highly expressed and was closely related to the prognosis of patients (Cai et al., 2018; Deng et al., 2018; Ghislin et al., 2012; Ren et al., 2019; Wang et al., 2020). Although it has been proved to be closely related to its occurrence and development in many different tumors, the role of PHF19 in CRC has not been studied.

In this study, the data from GEO, TCGA and Zhongshan cohort were used to compare the expression of PHF19 mRNA and protein in CRC. Subsequently, the relevant clinical data was used to analyze the correlation between PHF19 mRNA and protein and the patient’s clinicopathological factors; Kaplan–Meier (K–M) survival analysis and Cox regression were used to analyze the relationship between PHF19 and the patient’s long-term survival and related independent risk factors. Finally, bioinformatics analysis is used to find signal pathways related to the expression of PHF19. Using basic experiments, we studied the effect of PHF19 on the proliferation, invasion and metastasis of CRC in vitro and in vivo and verified the molecules that regulate the cell cycle and EMT process at the protein level.

## Materials and Methods

### Ethics statement

All of the study designs and test procedures were performed following the Helsinki Declaration II. The use of human tissue samples and clinical data was approved by the ethics committee of Zhongshan Hospital, Fudan University (2020-273R), and all patients in this study obtained an informed consent form for exemption.

### Gene expression analysis

Seven expression microarray series GSE21510 (Tsukamoto et al., 2011), GSE21815 (Iwaya et al., 2012), GSE9348 (Hong et al., 2010), GSE18105 (Matsuyama et al., 2010), GSE8671 (Sabates-Bellver et al., 2007), GSE44076 (Moreno et al., 2018) and GSE10715 (Galamb et al., 2008) containing CRC tumor and normal tissue were download from the Gene Expression Omnibus (GEO, https://www.ncbi.nlm.nih.gov/geo).

The cancer genome atlas (TCGA) CRC mRNA data and clinical information were downloaded from the UCSC Xena platform, which is a TCGA data analysis and download platform (Goldman et al., 2020). TCGA of colon adenocarcinoma and rectum adenocarcinoma (TCGA-COADREAD) RNAseq data contains 347 primary CRC samples and 56 normal tissue samples excluding patients with unknown age, gender, race, and TNM stage. Using seven GEO datasets and TCGA-COADREAD dataset, we compared the expression of PHF19 in CRC tissues and normal tissues.

### Tissue microarray and immunohistochemistry (IHC)

The tissue microarray of 83 pathologic diagnosed CRC tumor and adjacent normal tissue were obtained from Zhongshan Hospital, Fudan University. We obtained clinical information including age, sex, pathologic grade, tumor size, AJCC 7^th^ TNM stage, CA19-9, liver metastasis, and survival status. The immunohistochemistry of PHF19 protein expression level was performed using the anti-PHF19 antibody (GTX32787; GeneTex, Irvine, CA, USA, diluted 1:200). The interpretation of the results was carried out by two pathologists, who were unaware of the clinical information of the specimens. Each sample selected three typical visual fields under 200 magnification for evaluation. If the scores of a specimen were inconsistent, re-evaluate separately. The immunohistochemical score is calculated according to the staining intensity and the proportion of positively stained cells. The intensity of staining was scored as 0–3 points: zero (negative), one (weak), two (moderate), three (strong). The proportion of positively stained cells were used to describe the extend of staining: zero (none), one (0–25%), two (26–50%), three (51–75%), four (>75%). The scores of the two groups are multiplied to form the final immunohistochemical (IHC) score. IHC score less than three points is considered the low expression, more than three points is considered high expression. A paired t-test was used to compare the expression of PHF19 in the tumor and its adjacent normal tissues.

### Survival and clinical correlation analysis

The CRC patients were divided into two groups, high-expression group and low expression group, according to the median of PHF19 mRNA expression level or IHC score. The role of PHF19 in predicting OS were assessed by Kaplan–Meier (K–M) analysis. GSE38832, TCGA-COADREAD, and Zhongshan cohort were used to describe the relationship between PHF19 expression level and prognosis. The clinical information including age, sex, race, primary site, pathological grade, AJCC TNM stage, tumor size, CA19-9, CEA were extracted from the TCGA-COADREAD dataset and Zhongshan cohort. These information were used to perform clinical correlation analysis with PHF19. Then, using univariate and multivariate Cox regression analysis, we further verify whether PHF19 could be used as an independent prognostic factor for CRC patients.

### Co-expression analysis and GO/KEGG analysis

Using LinkedOmics (Vasaikar et al., 2018), an online analysis tool for gene co-expression, we analyzed the genes co-expressed with PHF19 in the TCGA-COADREAD dataset. A heat map was used to show typical genes related to PHF19 gene expression. Genes with correlation coefficient greater than 0.3 or lower than 0.3 are summarized and subjected to subsequent Gene Ontology (GO) and Kyoto Encyclopedia of Genes and Genomes (KEGG) pathway enrichment analysis.

### Gene set enrichment analysis (GSEA)

To further identify the enriched pathway of PHF19 furtherly, the CRC tissue sample was divided into two groups according to the median mRNA expression level of PHF19. We performed GSEA analysis (https://www.gsea-msigdb.org/gsea/index.jsp) of CRC tissue samples from the TCGA database to find the enriched pathway. Enrichment pathways with *P*-values less than 0.05 were considered statistically significant.

### Cell cultures, reagents and antibodies

The SW480 and HT-29 cells were purchased from the cell bank of Shanghai Institutes for Biological Sciences (Shanghai, China). SW480 were cultured in RPMI 1640 (Hyclone, Logan, TX, USA) and HT-29 were cultured in DMEM (Gibco, Brookyln, NY, USA) with 5% CO_2_ in 37 °C. All media were supplemented with 10% Fetal Bovine Serum (16000-044, Gibco, Brookyln, NY, USA) and 1% penicillin/streptomycin. The main antibodies including purchased from PHF19 (77271), Cyclin D1 (55506), E-cadherin (3195), N-cadherin (13116), β-catenin (8480), and Twist (46702) were purchased from Cell Signaling Technology (Beverly, MA, USA); H3K27me3 (ab6002) was purchased from Abcam (Cambridge, UK); CDK4 (11026-1-AP), CDK6 (14052-1-AP), Tubulin (11224-1-AP), and GADPH (10494-1-AP) were purchased from Proteintech (Wuhan, Hubei, China).

### Cell transfection

Cells were cultured in a six-well plate, and Opti-MEM was used to replace the conventional medium. The PHF19 plasmid (YouBio, Xian, China) and the control plasmid were transfected into cells using Lipofectamine 2000 (Life Technologies, Carlsbad, CA, USA), according to the operating instructions provided by the manufacturer. Lentiviruses that expressed PHF19 was purchased from Genechem (Shanghai, China) and transfected according to the manufacturer’s protocol.

### Western blot analysis

The cell lysate was separated by sodium dodecyl sulfate polyacrylamide gel electrophoresis and transferred to a polyvinylidene fluoride membrane. The membrane was then incubated with the primary antibody and then with the horseradish peroxidase-conjugated secondary antibody. Finally, chemiluminescence enhancement assay (Amersham Imager 600; GE, Schenectady, NY, USA) was used to detect protein expression.

### Cell proliferation and clone formation experiments

Approximately 2,000 SW480 and HT-29 cells per well were planted in 96-well plates, and the proliferation ability of the cells was tested according to the requirements of the manufacturer of the Cell Counting Kit-8 (Beyotime, China). Approximately 1,000 SW480 and HT-29 cells per well were planted in a 6-well plate. After two weeks of culture, it was fixed and stained with paraformaldehyde and crystal violet.

### Detection of cell cycle by flow cytometry

After harvesting the cells, the medium was removed by centrifugation, and then fixed with 80% ethanol overnight. The cells were taken out the next day and centrifuged. After half an hour of reaction in the staining solution, the cell cycle was detected by flow cytometry.

### Tumorigenesis in xenograft mouse model

BALB/c male nude mice aged 4–6 weeks were purchased from JSJ (Shanghai, China). SW480 cells (Lv-EV, Lv-PHF19) were resuspended in PBS at a density of 2 × 10^6^ and inoculated subcutaneously on nude mice (6 mice/group). Changes in tumor size were detected weekly. The mice were sacrificed on the 28th day to detect the size and volume of the tumor. Tumor volume was measured with a digital caliper and calculated it as 4π/3 × length/2 × width^2^ (mm).

### Migration and invasion assays

A 24-well Matrigel invasion chamber (Corning, NY, USA) was used to evaluate the invasion ability. SW480 (4 × 10^4^) and HT-29 cells (5 × 10^4^) were suspended in serum-free medium and added to the upper chamber. The medium containing 20% FBS was added to the lower chamber, and after 48 h of culture, the chamber was taken out and fixed and stained. The migration experiment operation was the same as the invasion experiment, except that Matrigel was not used.

### Statistical analysis

R software version 3.6.3 (https://www.r-project.org) and SPSS 20.0 from (IBM, Chicago, USA) were used to finish the statistical calculation. We used the Student’s *t-*test to compare the difference in the expression level of the PHF19 gene. Pearson Chi-square test was used to compare the clinical character with PHF19 expression level. The Cox proportional hazards regression model was used to confirm the independent prognostic factor from the TCGA database and Zhongshan cohort. The Kaplan-Meier method was used to estimate the OS of CRC patients according to the PHF19 mRNA expression level and IHC score. Paired and unpaired *t*-tests were used to compare the differences between the experimental group and the control group in cell and animal experiments. *P*-values were calculated by two-tailed, which less than 0.05 were considered to be statistically significant.

## Results

### PHF19 is overexpressed in CRC

**Figure 1:**
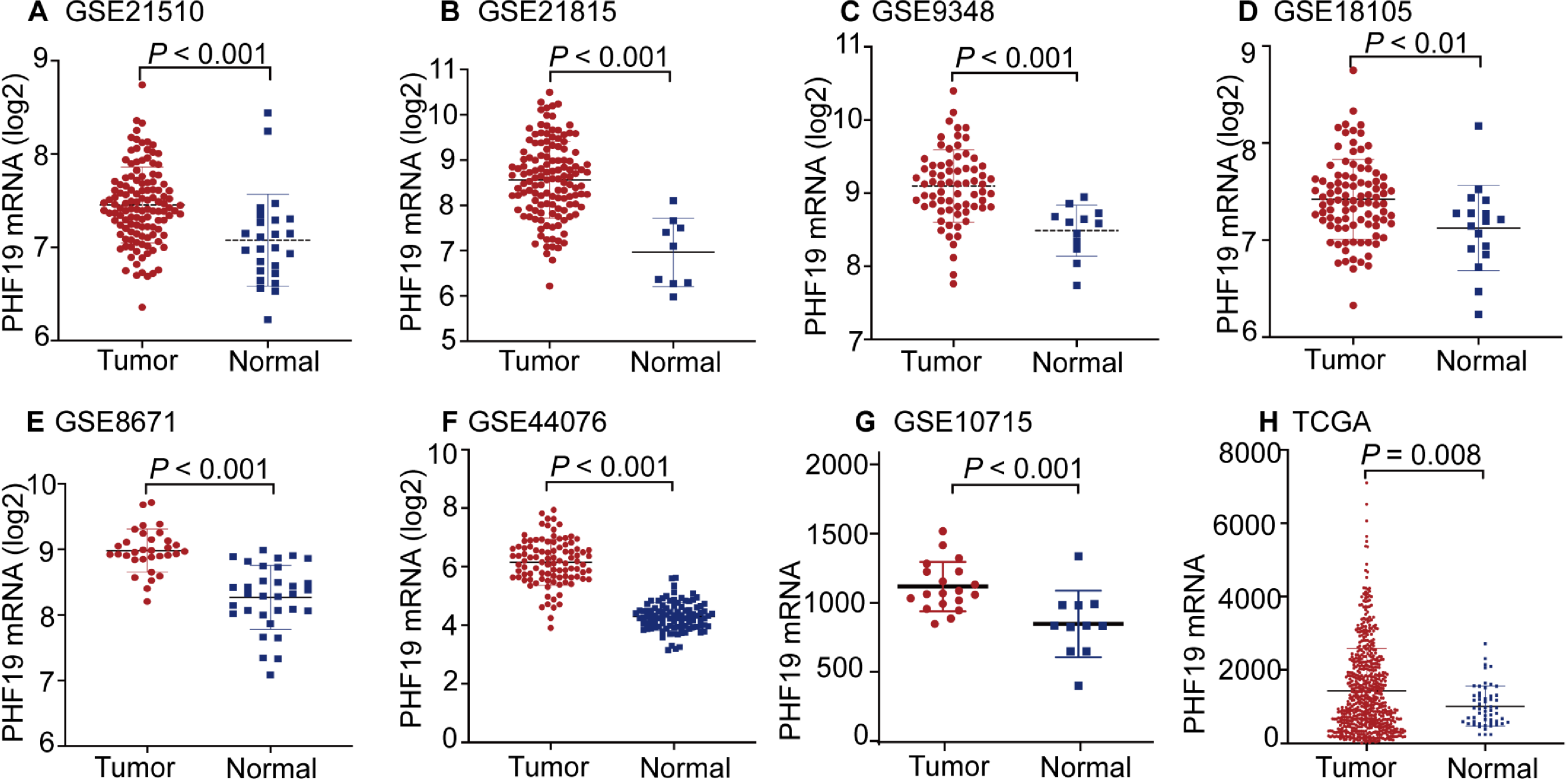
PHF19 mRNA expression levels between tumor and normal tissues in CRC patients in GEO database series and TCGA-COADREAD database. PHF19 mRNA expression levels between tumor and normal tissues in CRC patients in GEO database series including GSE21510 (A), GSE21815 (B), GSE9348 (C), GSE18105 (D), GSE8671 (E), GSE44076 (F), GSE10715 (G), and the TCGA-COADREAD database (H).

**Figure 2:**
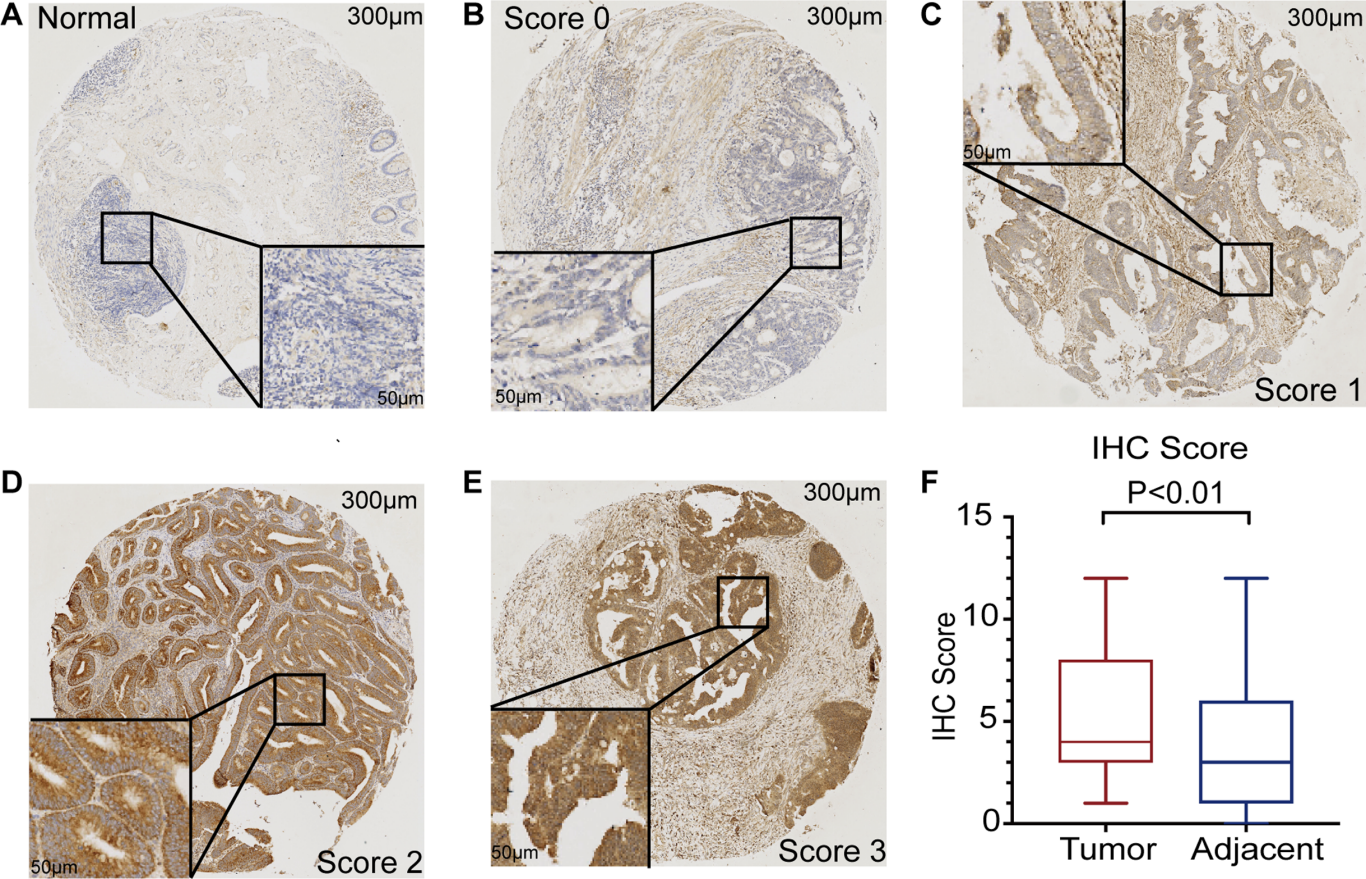
PHF19 protein expression level based on immunohistochemical assay using tissue microarray. PHF19 protein expression level based on immunohistochemical assay using tissue microarray containing 83 primary CRC cancer tissue (A–E). PHF19 IHC score between tumor and adjacent normal tissue in the Zhongshan cohort (F).

The mRNA expression of PHF19 was significantly overexpressed in GSE21510, GSE21815, GSE9348, GSE18105, GSE8671, GSE44076, GSE10715, and TCGA-COADREAD datasets (all *P* < 0.01, Fig. 1). To verify the expression of PHF19 protein in CRC, we analyzed 83 surgically resected paired CRC samples from the tissue microarrays (TMAs). Compared with paired adjacent normal tissues, PHF19 protein was overexpressed in CRC tissues (*P* < 0.01, Fig. 2F). Representative immunohistochemical stained specimens are shown in Figs. 2A–2E.

### Overexpression of PHF19 suggests a worse clinical outcome

**Figure 3:**
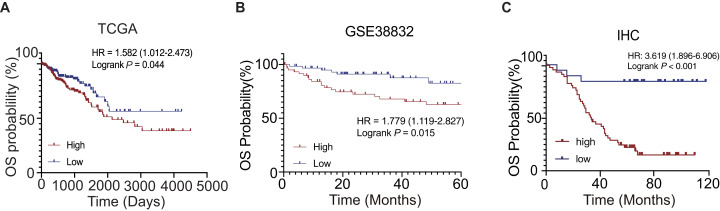
OS of CRC patients in different expression cohort: (A) TCGA database; (B) GSE38832; (C) IHC of the CRC patients in the Zhongshan cohort.

According to the clinical information of the TCGA dataset, we found that the PHF19 mRNA expression was closely related to the AJCC T stage (the depth of invasion, *P* = 0.026) and the CEA level (*P* < 0.001) (Table 1). We found that the PHF19 protein expression was closely related to the AJCC TNM stage (*P* = 0.041), AJCC M stage (*P* = 0.027) and liver metastasis (*P* = 0.049) according to the clinical information of Zhongshan cohort (Table 2). The results of K–M survival curves showed that the high expression of PHF19 mRNA (TCGA, *P* = 0.044; GSE38832, *P* = 0.015) and protein levels (IHC, *P* < 0.001) both predicted the poor prognosis of the patients (Fig. 3).

**Table 1:** Characteristics of CRC patients between PHF19 high and low cohort from the TCGA database.

Variables	PHF19 mRNA level		*P* value
	High (*n* = 173)	Low (*n* = 174)	
Age (median, years)	65.2	62	0.217
Gender			0.785
Male	93 (53.8)	91 (52.3)	
female	80 (46.2)	83 (47.7)	
Primary site			0.975
Colon	132 (76.3)	133 (76.4)	
Rectum	23 (13.3)	24 (13.8)	
Junction	18 (10.4)	17 (9.8)	
Race			0.185
White	139 (80.3)	131 (75.3)	
Black	31 (17.9)	34 (19.5)	
Other	3 (1.7)	9 (3.5)	
Histology type			0.244
adenomas	148 (85.5)	151 (86.8)	
Mucinous	25 (14.5)	23 (13.2)	
Stage			
I	21 (12.1)	31 (17.8)	0.402
II	66 (38.2)	59 (33.9)	
III	59 (34.1)	59 (33.9)	
IV	27(15.6)	25 (14.4)	
AJCC T stage			0.026
T1	7 (4.0)	5 (2.9)	
T2	17 (9.8)	31 (17.8)	
T3	118 (68.2)	122 (70.1)	
T4	31 (17.9)	16 (9.2)	
AJCC N stage			0.867
N0	89(51.4)	94(54.0)	
N1	51(29.5)	50(28.7)	
N2	33(19.1)	30(17.2)	
AJCC M stage			
M0	123 (83.1)	117 (82.4)	0.872
M1	25 (16.9)	25 (17.6)	
CEA (median, ng/ml)	91.1	40.5	<0.001
Lymph invasion			0.495
Yes	52 (33.1)	47 (29.6)	
No	105 (66.9)	112 (70.4)	
Vein invasion			0.077
Yes	31 (20.0)	16 (12.2)	
No	124 (80.0)	115 (87.8)	

**Table 2:** Characteristics of CRC patients between PHF19 high and low cohort from the Zhongshan cohort.

Variables	PHF19 protein level		*P* value
	High (*n* = 59)	Low (*n* = 24)	
Age (median, years)	59.5	54.4	0.145
Gender			0.81
Male	32 (54.2)	12 (50.0)	
female	27 (45.8)	12 (50.0)	
Grade			0.536
High and moderate	47 (79.7)	21 (87.5)	
Poorly	12 (20.3)	3 (12.5)	
Stage			0.041
I	4 (6.8)	5 (20.8)	
II	18 (30.5)	11 (45.8)	
III	18 (30.5)	6 (25.0)	
IV	19 (32.2)	2 (8.3)	
AJCC T stage			0.77
T1-T2	12 (20.3)	6 (25.0)	
T3-T4	47 (79.7)	18 (75.0)	
AJCC N stage			0.454
N0	35 (59.3)	17 (70.8)	
N1-N2	24 (40.7)	7 (29.2)	
AJCC M stage			0.027
M0	40 (67.8)	22 (91.7)	
M1	19 (32.2)	2 (8.3)	
Liver metastasis			0.049
Yes	17 (28.8)	2 (8.3)	
No	42 (71.2)	22 (91.7)	
Tumor size			0.335
> 5	22 (37.9)	12 (50.0)	
≤ 5	36 (62.1)	12 (50.0)	
CA19-9			0.702
Positive	30 (66.7)	13 (59.1)	
Negative	12 (26.7)	8 (36.4)	
NA	3 (6.7)	1 (4.5)	

### PHF19 is an independent prognostic factor for CRC patients

Using the clinical data from the TCGA database, we found that the expression of PHF19 mRNA (*P* = 0.046), age (*P* = 0.001), and AJCC TNM stage (*P* < 0.001) were considered to be independent risk factors for the prognosis of CRC patients (Table 3). Using the clinical data of TMAs’ patients from Zhongshan cohort, we found that the expression of PHF19 protein (*P* = 0.025), and AJCC TNM stage (*P* < 0.001) were considered to be independent risk factors for the prognosis of CRC patients (Table 4).

**Table 3:** Univariate and multivariate Cox regression analyses of OS for CRC patients in the TCGA database.

Variables	Univariate analysis			Multivariate analysis		
	HR	95% CI	*P* value	HR	95% CI	*P* value
age	1.972	[1.225–3.174]	0.005	2.303	[1.422–3.373]	0.001
sex	0.791	[0.505–1.239]	0.306			
race	1.146	[0.689–1.906]	0.600			
T stage	1.599	[1.057–2.420]	0.026			0.458
N stage	1.410	[1.070–1.859]	0.015			0.641
M stage	0.966	[0.718–1.300]	0.819			
TNM stage	1.850	[1.421–2.408]	<0.001	2.011	[1.535–2.635]	<0.001
Primary site	0.598	[0.296–1.206]	0.151			
PHF19	1.604	[1.008–2.553]	0.046	1.603	[1.008–2.549]	0.046

**Table 4:** Univariate and multivariate Cox regression analyses of OS for CRC patients in the Zhongshan cohort.

Variables	Univariate analysis			Multivariate analysis		
	HR	95% CI	*P* value	HR	95% CI	*P* value
age	1.113	[0.597–2.073]	0.736			
sex	1.282	[0.696–2.366]	0.427			
grade	2.241	[1.141–4.4]	0.455			
T stage	1.503	[0.665–3.397]	0.327			
N stage	1.751	[0.948–3.233]	0.07			
M stage	12.519	[5.880-26.652]	<0.001			0.240
TNM stage	4.497	[2.784–7.265]	<0.001	3.850	[2.375–6.241]	<0.001
Tumor size	0.614	[0.238–1.152]	0.129			
PHF19	7.401	[2.274–24.09]	0.001	4.031	[1.202–13.516]	0.025
Liver metastasis	11.826	[5.687–24.594]	<0.001			0.117
CA19-9	0.417	[0.214–0.811]	0.01			0.261

### Co-expression analysis and GO/KEGG enrichment

**Figure 4:**
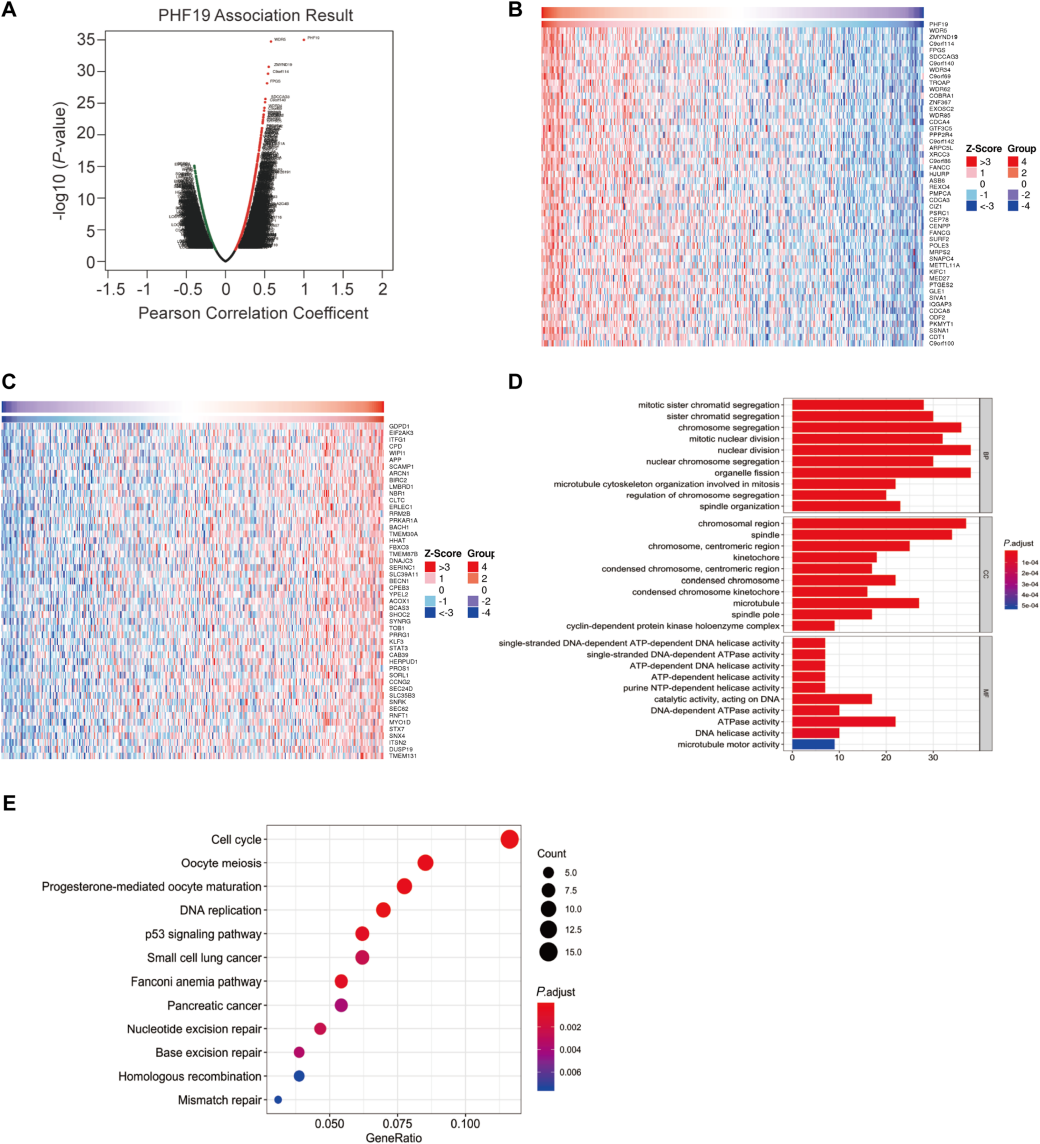
Co-expression analysis of PHF19 using LinkOmics. Co-expression analysis of PHF19 using LinkOmics. Highly correlated genes were identified by Pearson test in COADREAD cohort (A). Heat maps showing top 50 genes correlated with PHF19 in COADREAD. Red indicates positive scores and blue indicates negative scores (B, C). Enrichment of GO annotations and KEGG pathways of PHF19 in the COADREAD cohort (D, E).

Using LinkedOmics platform to perform co-expression analysis for PHF19, the PHF19 association results were shown in Fig. 4A. Among the co-expressed genes of PHF19, we found that 275 genes were positively correlated with PHF19, and 83 genes were negatively correlated with PHF19, and their correlation coefficients | r | all exceed 0.3. The heatmap showed the top 50 positively and negatively co-expressed genes of PHF19 (Figs. 4B, 4C).

Summarizing the genes whose correlation coefficient | r | was greater than 0.3, and we used these genes to further perform GO enrichment analysis and KEGG pathway analysis (Figs. 4D, 4E). GO enrichment analysis showed us that PHF19 is mainly enriched in biological processes related to chromosome activation in biological processes, such as chromosome segregation, nuclear division, etc. And the KEGG pathway analysis indicated that PHF19 was most enriched in 12 pathways, such as cell cycle, DNA replication, p53 signaling, small lung cancer, pancreatic cancer.

### GSEA analysis between high and low PHF19 expression

**Figure 5:**
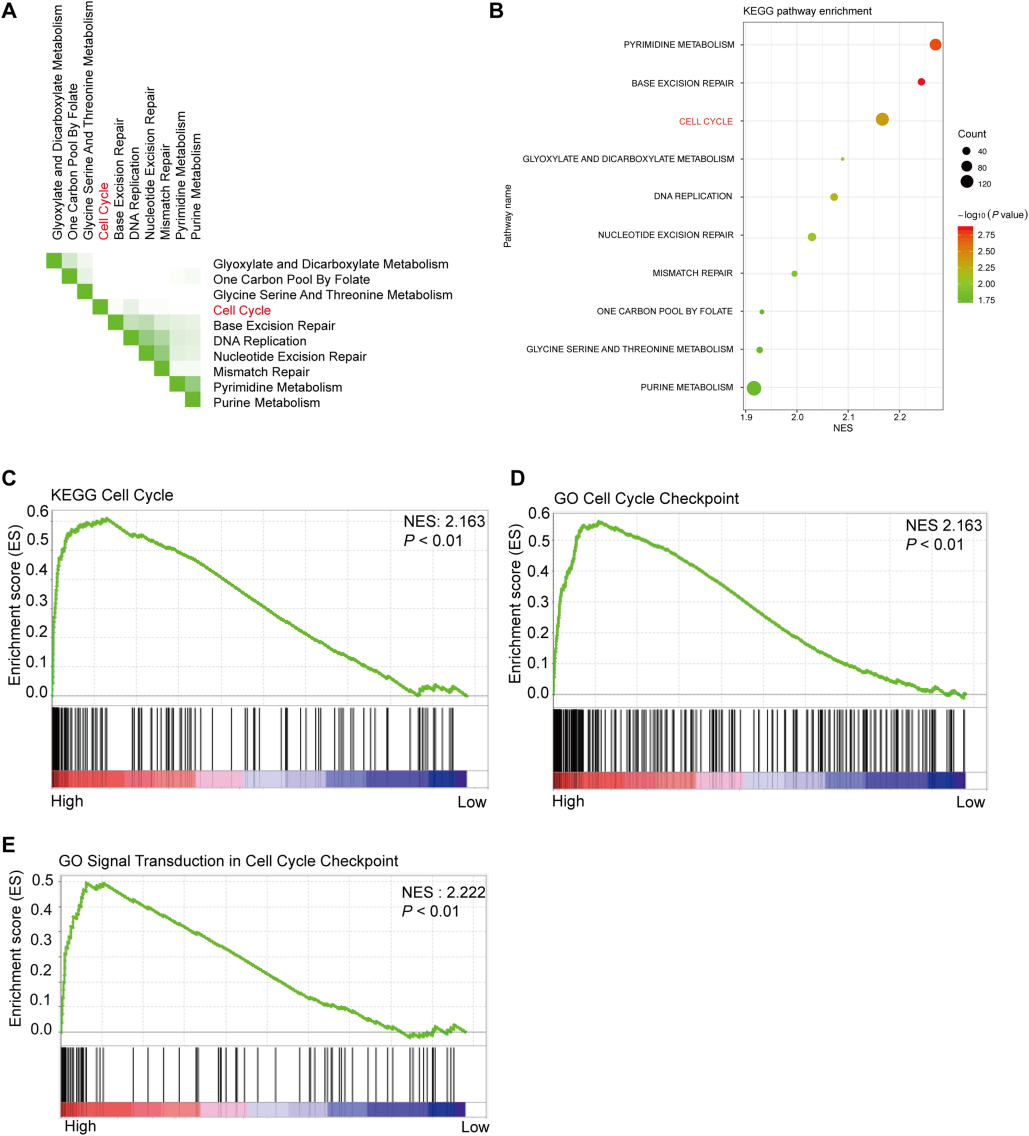
High PHF19 expression positively correlates with Cell Cycle in CRC patients. High PHF19 expression positively correlates with Cell Cycle in CRC patients. Top ten signaling pathway in CRC tissue based on GSEA (A). Enrichment of KEGG pathways by GSEA using the TCGA database (B). The enrichment plot shows that GSEA analysis of genes involved in cell cycle (C, D, E).

Using 347 CRC tissue samples from the TCGA database, we divided it into two groups: high PHF19 and low PHF19. Then among the top 10 pathways shown in Figs. 5A and 5B, cell cycle-related pathways were still in the top ranking. The results of GSEA indicated that high expression of PHF19 was closely related to cell cycle regulation (*P* < 0.01, Figs. 5C–5E).

### PHF19 promoted the proliferation abilities of CRC cells in vitro and in vivo

**Figure 6:**
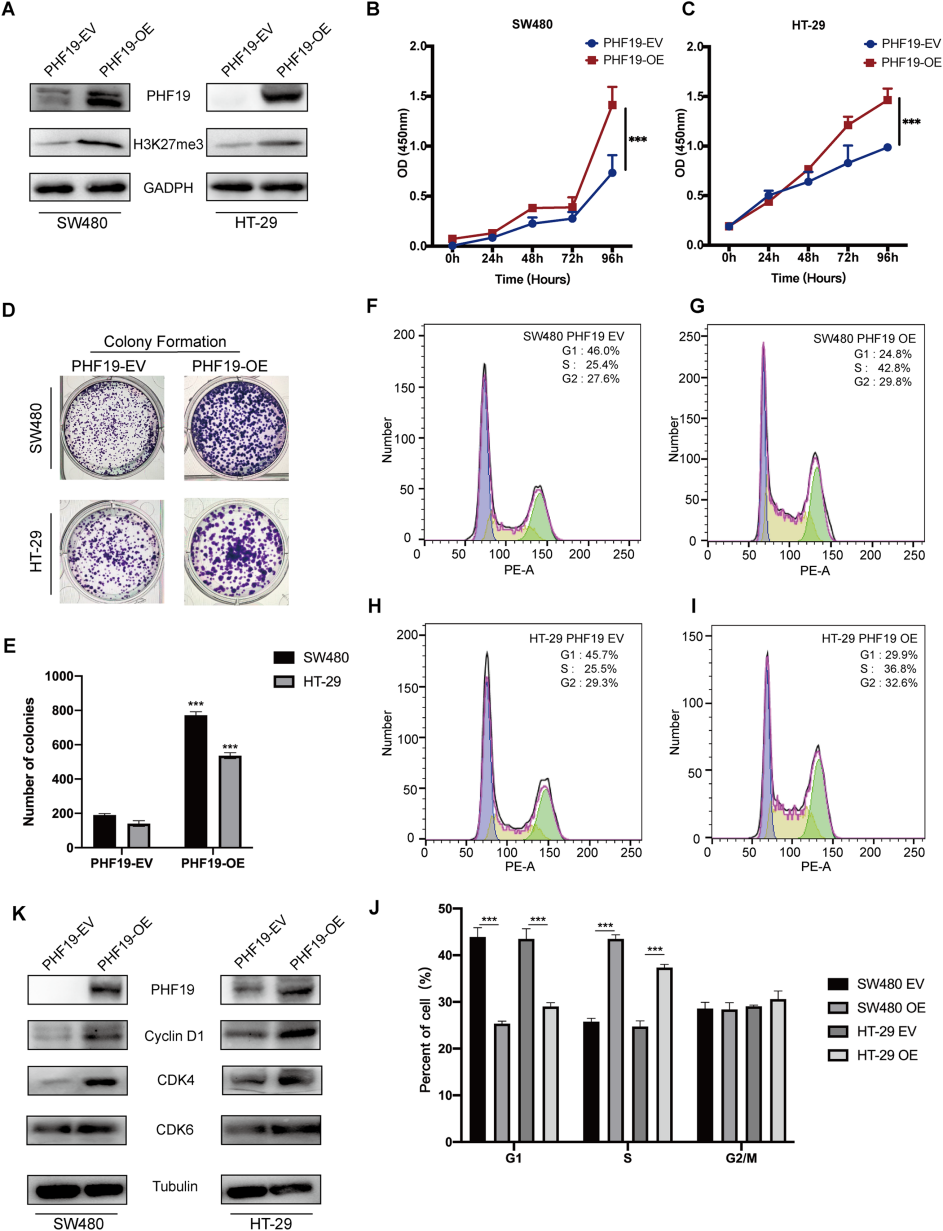
Overexpression of PHF19 promoted CRC cell proliferation in vitro. Overexpression of PHF19 promoted CRC cell proliferation in vitro. PHF19 and H3K27me3 expression in SW480 and HT-29 cell lines transfected with PHF19-EV and PHF19-OE plasmids (A). CCK-8 and clone formation experiments were used to judge the changes in the proliferation ability of CRC cells (B–E). Flow cytometry was used to detect cell cycle changes after PHF19 overexpression (F–J). Western blot was used to detect the expression of cell cycle related proteins (K). (***means *P* < 0.001).

In order to verify the role of PHF19 in CRC, we used plasmids to overexpress PHF19. Compared with the control group, Western blot found that the expression levels of PHF19 and H3K27me3 in SW480 and HT-29 were increased after overexpression of PHF19 (Fig. 6A). CCK-8 and clone formation experiments were used to detect the changes in the proliferation ability of SW480 and HT-29 after overexpression of PHF19. As shown in Figs. 6B, 6C, overexpression of PHF19 significantly enhanced the proliferation ability of SW480 and HT-29.

Next, flow cytometry was used to detect the cell cycle, and it was found that tumor cells accelerated the transition from G1 phase to S phase (Fig. 6D); Western blot showed that the expression of Cyclin D1, CDK4, and CDK6 protein was up-regulated, indicating that PHF19 upregulated the expression of Cyclin D1, CDK4, and CDK6 to promote the proliferation of SW480 and HT-29 (Fig. 6E).

**Figure 7:**
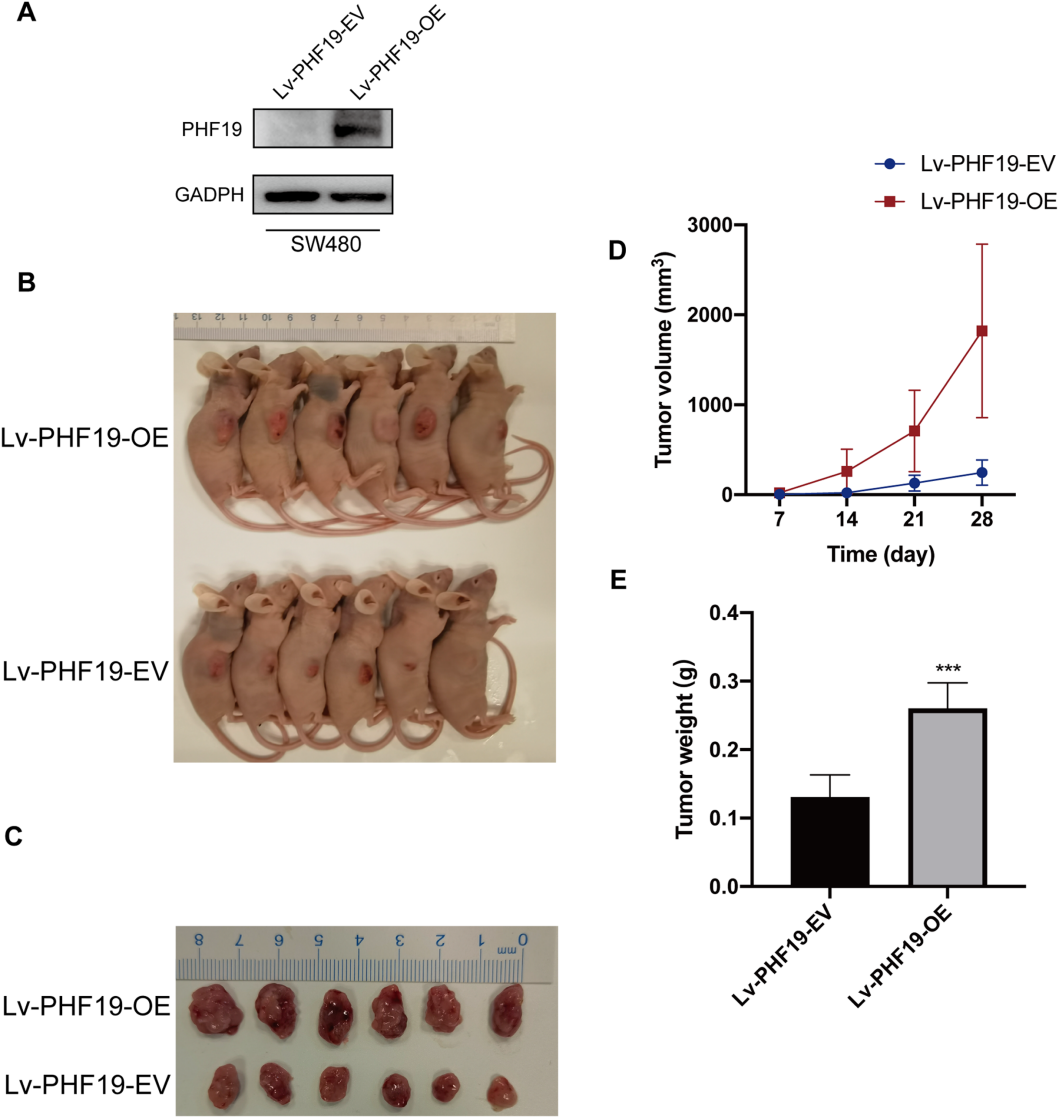
Overexpression of PHF19 promoted tumor formation in vivo. Overexpression of PHF19 promoted tumor formation in vivo. PHF19 expression in SW480 cell lines transfected with Lv-PHF19-EV and Lv-PHF19-OE (A). Images of representative mice were shown (B, C). Tumor size and volume changes were shown through statistical graphs (D, E). (***means *P* < 0.001).

In addition, in order to further verify the role of PHF19 in CRC, we constructed SW480 that stably overexpresses PHF19. Through subcutaneous tumor formation experiments in nude mice, we found that compared with the control group, mice in the PHF19 overexpression group obtained larger tumor tissues and heavier tumors after 28 days (Fig. 7).

### PHF19 promoted the migration and invasion abilities of CRC cells in vitro

**Figure 8:**
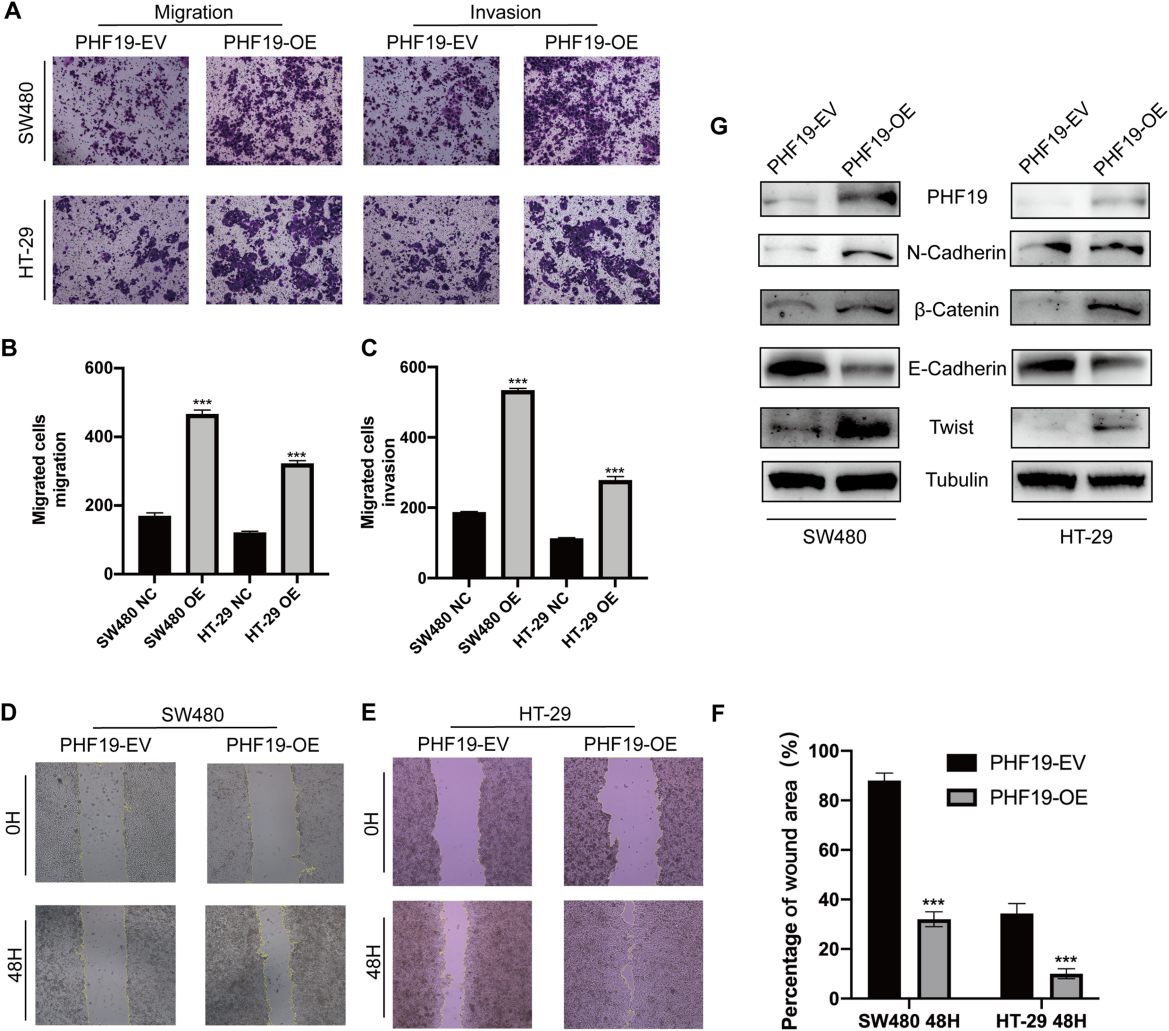
Overexpression of PHF19 promoted CRC cell migration and invasion by inducing EMT in vitro. Overexpression of ****PHF19 promoted CRC cell migration and invasion by inducing EMT in vitro. Transwell was used to examine cell migration and invasion in SW480 and HT-29 transfected with PHF19 overexpression plasmid (A–C). Wound healing assay was used to examine cell migration in SW480 and HT-29 transfected with PHF19 overexpression plasmid (D–F). Western blot was used to detect the expression of EMT related proteins (G). (***means *P* < 0.001).

Clinical correlation analysis showed that the high expression of PHF19 was closely related to the metastatic phenotype of CRC. For this reason, we used Transwell experiment and wound experiment to verify the effect of overexpression of PHF19 on the invasion and migration ability of CRC cells. Overexpression of PHF19 significantly increased the invasion and migration ability of CRC cells, and the number of cells passing through the membrane into the lower chamber was significantly increased compared to the control group (Figs. 8A, 8B). Wound healing experiments found that the healing speed was significantly accelerated after overexpression of PHF19 (Fig. 8C).

Western blot showed that the key proteins of EMT, β-catenin, Twist, and N-cadherin, were up-regulated, and E-cadherin was down-regulated, indicating that PHF19 promotes tumor cell invasion and metastasis by regulating EMT-related molecules.

## Discussion

CRC is one of the most common cancer in the world, the treatment and rehabilitation process brought a lot of pain to the patients (Bray et al., 2018; Weitz et al., 2005). Although the diagnosis and treatment technology has made great progress, how to correctly judge the prognosis of CRC patients still needs further research (Chen et al., 2020; Molgora et al., 2020). Therefore, it is very important to further clarify the underlying mechanism of CRC, especially in the molecular markers related to prognosis. In recent years, more and more attention has been paid to the mechanism of PHF19 in tumorigenesis and development (Cai et al., 2018). Among different tumors, PHF19 was found to be significantly up-regulated in tumor tissues, which is considered to be closely related to the poor prognosis of patients, such as gastric cancer, melanoma, liver cancer, glioblastoma (Cai et al., 2018; Deng et al., 2018; Ghislin et al., 2012; Wang et al., 2020). In the meanwhile, the role of PHF19 in tumors has been confirmed to be closely related to the process of tumor cell proliferation and metastasis (Cai et al., 2018; Ghislin et al., 2012). However, the mechanism of PHF19 in CRC has not been confirmed accordingly, so our research will explain the role of the PHF19 in CRC.

In this study, using seven GEO datasets and TCGA-COADREAD dataset, we found that at the mRNA level, compared with normal tissues, PHF19 mRNA was significantly upregulated in CRC tissues. Then the IHC results of TMA using 83 paired CRC tumors from Zhongshan cohort showed us that the PHF19 protein was also upregulated in the CRC sample. And using prognostic data from three cohorts: GSE38832, TCGA, and Zhongshan cohort, we found that high expression of PHF19 is closely related to the poor prognosis of patients regardless of the mRNA level or the protein level. This further confirmed the possibility of PHF19 as a prognostic molecule for CRC. The results of correlation analysis based on clinicopathological data indicate that PHF19 mRNA was closely related to the depth of tumor invasion, and the relatively higher expression of PHF19 mRNA in the later T stage suggested that it might be related to tumor progression. Carcinoembryonic antigen (CEA) has been used as a tumor biomarker in gastrointestinal cancer for decades, but its effect in predicting the survival rate of patients with CRC is still not perfect (Locker et al., 2006; Primrose et al., 2014). Our results using TCGA dataset showed that high expression of PHF19 mRNA was positively correlated with the level of CEA, suggesting that PHF19 may play a synergistic effect with CEA in judging the survival and prognosis of patients.

Then we used Zhongshan cohort to verify the relationship between PHF19 protein expression and clinicopathological data, we found that high PHF19 protein expression indicated worse tumor stage and more likely tumor metastasis, especially liver metastases. Metastasis is an important factor affecting the prognosis of CRC (Teng et al., 2020). The most common colorectal metastasis is liver metastasis, accounting for 20–34% (Li et al., 2020). A more in-depth and thorough elucidation of the mechanism of CRC liver metastasis is a goal that researchers have been working hard to achieve. According to our results, we supposed that PHF19 may affect tumor progression by affecting the metastasis ability of CRC. Using univariate and multivariate Cox regression, we found that the expression of PHF19 can be used as an independent prognostic factor for patients with CRC, no matter at the RNA level or the protein level. This also means that PHF19 is an excellent prognostic indicator for patients with CRC. Combining PHF19 with other independent prognostic factors such as age and tumor TNM staging can build a good prognostic judgment model for CRC patients.

PHF19 is generally considered to play an important role in the process of chromosome activation (Brien et al., 2012; Sauvageau & Sauvageau, 2010). Recent studies have shown that PHF19 can promote the malignant proliferation of tumors by regulating cell circle and cell invasion and metastasis (Cai et al., 2018; Deng et al., 2018; Ren et al., 2019). PHF19 activated PRC2 and promoted the spread of H3K27me3, thereby enhancing its promotion of tumor formation (Ren et al., 2019). Due to this activation, it improved the sensitivity of tumors to PRC2 inhibitors. In melanoma, Akt has been found to play a biological effect as an upstream regulatory molecule of PHF19 (Ghislin et al., 2012). Through the regulation of AKT, tumor cells could switch between cell proliferation and invasion phenotypes (Ghislin et al., 2012). In our research, CCK-8 and clone formation experiments confirmed that PHF19 could promote the malignant proliferation of CRC cells through its high expression, which is consistent with the results of previous studies in gastric cancer (Wang et al., 2020). Flow cytometry was used to detect cell cycle changes after PHF19 transfection. Compared with control group, the proportion of S phase increased, and the ratio of G1 phase decreased, which means that PHF19 accelerated the G1 to S phase transition CRC. In glioma, similar conclusions had also been fully experimentally confirmed (Deng et al., 2018). Further in vivo experimental results confirmed that PHF19 could indeed promote the proliferation of CRC. As a transcription factor that regulates chromatin function, PHF19 has also been proven to regulate tumor EMT progression in a variety of tumor studies (Abdelfettah et al., 2020; Deng et al., 2018; Wang et al., 2020). PHF19 increases the expression of β-catenin, N-cadherin, Snail and other molecules in gliomas, inhibits the expression of E-cadherin, and promotes the occurrence of metastatic malignant phenotypes of gliomas (Deng et al., 2018). PHF19 interacted with the components of β-catenin inhibitor in the intercellular substance, inhibiting the decomposition of β-catenin, and promoted the signal transduction of β-catenin/T cells, thereby increasing the level of downstream IL-6 and promoting the movement of liver cancer cells (Cai et al., 2018). Therefore, using corresponding functional experiments, compared with the control group, the invasion of PHF19 overexpression group became stronger and it was easier to migrate to distant places. It also promoted the up-regulation of EMT-related proteins N-cadherin, β-catenin, and Twist, leading to down-regulation of E-cadherin, further confirming that PHF19 enhances the invasion of CRC by accelerating the progress of the EMT.

In conclusion, our study revealed for the first time that the expression of the PHF19 gene was up-regulated in CRC tissues and was closely related to tumor progression and the prognosis of patients with CRC and could be an independent prognostic factor. Besides, we found that the oncogene effect of PHF19 in CRC is very likely to be achieved by affecting tumor progression and regulating the cell cycle. PHF19 could increase the cell proliferation ability by increasing the expression of Cyclin D1, CDK4 and CDK6 to accelerate the transition of the cell cycle G1-S phase. PHF19 could reduce the expression of E-cadherin, increased the expression of N-cadherin, β-catenin, and Twist, and then promoted the EMT process to enhance the ability of metastasis. Therefore, our research proved that PHF19 could be a new biomarker for CRC, providing new evidence for the occurrence and development of CRC.

## Supplemental Information

10.7717/peerj.11551/supp-1 Supplemental Information 1 Raw data.

## Additional Information and Declarations

### Data Availability

The following information was supplied regarding data availability: The raw measurements are available in the Supplemental Files. PHF19 raw data are available at TCGA: https://portal.gdc.cancer.gov/genes/ENSG00000119403. Additional data from seven expression microarray series containing CRC tumor and normal tissue are available at NCBI GEO: GSE21510, GSE21815, GSE9348, GSE18105, GSE8671, GSE44076and GSE10715.

## References

[ref-1] Abdelfettah S, Boulay G, Dubuissez M, Spruyt N, Garcia SP, Rengarajan S, Loison I, Leroy X, Rivera MN, Leprince D (2020). hPCL3S promotes proliferation and migration of androgen-independent prostate cancer cells. Oncotarget.

[ref-2] Ballare C, Lange M, Lapinaite A, Martin GM, Morey L, Pascual G, Liefke R, Simon B, Shi Y, Gozani O, Carlomagno T, Benitah SA, Di Croce L (2012). Phf19 links methylated Lys36 of histone H3 to regulation of polycomb activity. Nature Structural & Molecular Biology.

[ref-3] Bray F, Ferlay J, Soerjomataram I, Siegel RL, Torre LA, Jemal A (2018). Global cancer statistics 2018: GLOBOCAN estimates of incidence and mortality worldwide for 36 cancers in 185 countries. CA: A Cancer Journal for Clinicians.

[ref-4] Brien GL, Gambero G, O’Connell DJ, Jerman E, Turner SA, Egan CM, Dunne EJ, Jurgens MC, Wynne K, Piao L, Lohan AJ, Ferguson N, Shi X, Sinha KM, Loftus BJ, Cagney G, Bracken AP (2012). Polycomb PHF19 binds H3K36me3 and recruits PRC2 and demethylase NO66 to embryonic stem cell genes during differentiation. Nature Structural & Molecular Biology.

[ref-5] Cai Z, Qian ZY, Jiang H, Ma N, Li Z, Liu LY, Ren XX, Shang YR, Wang JJ, Li JJ, Liu DP, Zhang XP, Feng D, Ni QZ, Feng YY, Li N, Zhou XY, Wang X, Bao Y, Zhang XL, Deng YZ, Xie D (2018). hPCL3s promotes hepatocellular carcinoma metastasis by activating β-Catenin signaling. Cancer Research.

[ref-6] Chen EX, Jonker DJ, Loree JM, Kennecke HF, Berry SR, Couture F, Ahmad CE, Goffin JR, Kavan P, Harb M, Colwell B, Samimi S, Samson B, Abbas T, Aucoin N, Aubin F, Koski SL, Wei AC, Magoski NM, Tu D, O’Callaghan CJ (2020). Effect of combined immune checkpoint inhibition vs best supportive care alone in patients with advanced colorectal cancer: the canadian cancer trials group CO.26 study. JAMA Oncology.

[ref-7] Deng Q, Hou J, Feng L, Lv A, Ke X, Liang H, Wang F, Zhang K, Chen K, Cui H (2018). PHF19 promotes the proliferation, migration, and chemosensitivity of glioblastoma to doxorubicin through modulation of the SIAH1/β-catenin axis. Cell Death & Disease.

[ref-8] Galamb O, Sipos F, Solymosi N, Spisak S, Krenacs T, Toth K, Tulassay Z, Molnar B (2008). Diagnostic mRNA expression patterns of inflamed, benign, and malignant colorectal biopsy specimen and their correlation with peripheral blood results. Cancer Epidemiology Biomarkers & Prevention.

[ref-9] Ghislin S, Deshayes F, Middendorp S, Boggetto N, Alcaide-Loridan C (2012). PHF19 and Akt control the switch between proliferative and invasive states in melanoma. Cell Cycle.

[ref-10] Goldman MJ, Craft B, Hastie M, Repecka K, McDade F, Kamath A, Banerjee A, Luo Y, Rogers D, Brooks AN, Zhu J, Haussler D (2020). Visualizing and interpreting cancer genomics data via the Xena platform. Nature Biotechnology.

[ref-11] Hong Y, Downey T, Eu KW, Koh PK, Cheah PY (2010). A ‘metastasis-prone’ signature for early-stage mismatch-repair proficient sporadic colorectal cancer patients and its implications for possible therapeutics. Clinical & Experimental Metastasis.

[ref-12] Iwaya T, Yokobori T, Nishida N, Kogo R, Sudo T, Tanaka F, Shibata K, Sawada G, Takahashi Y, Ishibashi M, Wakabayashi G, Mori M, Mimori K (2012). Downregulation of miR-144 is associated with colorectal cancer progression via activation of mTOR signaling pathway. Carcinogenesis.

[ref-13] Li Y, Liu W, Zhao L, Güngör C, Xu Y, Song X, Wang D, Zhou Z, Zhou Y, Li C, Pei Q, Tan F, Pei H (2020). Nomograms predicting overall survival and cancer-specific survival for synchronous colorectal liver-limited metastasis. Journal of Cancer.

[ref-14] Locker GY, Hamilton S, Harris J, Jessup JM, Kemeny N, Macdonald JS, Somerfield MR, Hayes DF, Bast RC, Asco (2006). ASCO, 2006 update of recommendations for the use of tumor markers in gastrointestinal cancer. Journal of Clinical Oncology.

[ref-15] Matsuyama T, Ishikawa T, Mogushi K, Yoshida T, Iida S, Uetake H, Mizushima H, Tanaka H, Sugihara K (2010). MUC12 mRNA expression is an independent marker of prognosis in stage II and stage III colorectal cancer. International Journal of Cancer.

[ref-16] Molgora M, Esaulova E, Vermi W, Hou J, Chen Y, Luo J, Brioschi S, Bugatti M, Omodei AS, Ricci B, Fronick C, Panda SK, Takeuchi Y, Gubin MM, Faccio R, Cella M, Gilfillan S, Unanue ER, Artyomov MN, Schreiber RD, Colonna M (2020). TREM2 modulation remodels the tumor myeloid landscape enhancing anti-PD-1 immunotherapy. Cell.

[ref-17] Moreno V, Alonso MH, Closa A, Valles X, Diez-Villanueva A, Valle L, Castellvi-Bel S, Sanz-Pamplona R, Lopez-Doriga A, Cordero D, Sole X (2018). Colon-specific eQTL analysis to inform on functional SNPs. British Journal of Cancer.

[ref-18] Primrose JN, Perera R, Gray A, Rose P, Fuller A, Corkhill A, George S, Mant D, Investigators FT (2014). Effect of 3 to 5 years of scheduled CEA and CT follow-up to detect recurrence of colorectal cancer: the FACS randomized clinical trial. JAMA.

[ref-19] Qin S, Guo Y, Xu C, Bian C, Fu M, Gong S, Min J (2013). Tudor domains of the PRC2 components PHF1 and PHF19 selectively bind to histone H3K36me3. Biochemical and Biophysical Research Communications.

[ref-20] Ren Z, Ahn JH, Liu H, Tsai YH, Bhanu NV, Koss B, Allison DF, Ma A, Storey AJ, Wang P, Mackintosh SG, Edmondson RD, Groen RWJ, Martens AC, Garcia BA, Tackett AJ, Jin J, Cai L, Zheng D, Wang GG (2019). PHF19 promotes multiple myeloma tumorigenicity through PRC2 activation and broad H3K27me3 domain formation. Blood.

[ref-21] Roncucci L, Mariani F (2015). Prevention of colorectal cancer: how many tools do we have in our basket?. European Journal of Internal Medicine.

[ref-22] Sabates-Bellver J, Van der Flier LG, de Palo M, Cattaneo E, Maake C, Rehrauer H, Laczko E, Kurowski MA, Bujnicki JM, Menigatti M, Luz J, Ranalli TV, Gomes V, Pastorelli A, Faggiani R, Anti M, Jiricny J, Clevers H, Marra G (2007). Transcriptome profile of human colorectal adenomas. Molecular Cancer Research.

[ref-23] Sauvageau M, Sauvageau G (2010). Polycomb group proteins: multi-faceted regulators of somatic stem cells and cancer. Cell Stem Cell.

[ref-24] Teng S, Li YE, Yang M, Qi R, Huang Y, Wang Q, Zhang Y, Chen S, Li S, Lin K, Cao Y, Ji Q, Gu Q, Cheng Y, Chang Z, Guo W, Wang P, Garcia-Bassets I, Lu ZJ, Wang D (2020). Tissue-specific transcription reprogramming promotes liver metastasis of colorectal cancer. Cell Research.

[ref-25] Tsukamoto S, Ishikawa T, Iida S, Ishiguro M, Mogushi K, Mizushima H, Uetake H, Tanaka H, Sugihara K (2011). Clinical significance of osteoprotegerin expression in human colorectal cancer. Clinical Cancer Research.

[ref-26] Vasaikar SV, Straub P, Wang J, Zhang B (2018). LinkedOmics: analyzing multi-omics data within and across 32 cancer types. Nucleic Acids Research.

[ref-27] Wang H, Xu P, Sun G, Lv J, Cao J, Xu Z (2020). Downregulation of PHF19 inhibits cell growth and migration in gastric cancer. Scandinavian Journal of Gastroenterology.

[ref-28] Wang S, Robertson GP, Zhu J (2004). A novel human homologue of Drosophila polycomblike gene is up-regulated in multiple cancers. Gene.

[ref-29] Weitz J, Koch M, Debus J, Höhler T, Galle PR, Büchler MW (2005). Colorectal cancer. The Lancet.

[ref-30] Zhang L, Li Z, Skrzypczynska KM, Fang Q, Zhang W, O’Brien SA, He Y, Wang L, Zhang Q, Kim A, Gao R, Orf J, Wang T, Sawant D, Kang J, Bhatt D, Lu D, Li CM, Rapaport AS, Perez K, Ye Y, Wang S, Hu X, Ren X, Ouyang W, Shen Z, Egen JG, Zhang Z, Yu X (2020). Single-cell analyses inform mechanisms of myeloid-targeted therapies in colon cancer. Cell.

